# *Clostridium botulinum *group III: a group with dual identity shaped by plasmids, phages and mobile elements

**DOI:** 10.1186/1471-2164-12-185

**Published:** 2011-04-12

**Authors:** Hanna Skarin, Therese Håfström, Josefina Westerberg, Bo Segerman

**Affiliations:** 1 Department of Bacteriology, National Veterinary Institute (SVA), Uppsala, Sweden; 2 Department of Biomedical Sciences and Veterinary Public Health, Swedish University of Agricultural Sciences (SLU), Uppsala, Sweden

## Abstract

**Background:**

*Clostridium botulinum *strains can be divided into four physiological groups that are sufficiently diverged to be considered as separate species. Here we present the first complete genome of a *C. botulinum *strain from physiological group III, causing animal botulism. We also compare the sequence to three new draft genomes from the same physiological group.

**Results:**

The 2.77 Mb chromosome was highly conserved between the isolates and also closely related to that of *C. novyi*. However, the sequence was very different from the human *C. botulinum *group genomes. Replication-directed translocations were rare and conservation of synteny was high. The largest difference between *C. botulinum *group III isolates occurred within their surprisingly large plasmidomes and in the pattern of mobile elements insertions. Five plasmids, constituting 13.5% of the total genetic material, were present in the completed genome. Interestingly, the set of plasmids differed compared to other isolates. The largest plasmid, the botulinum-neurotoxin carrying prophage, was conserved at a level similar to that of the chromosome while the medium-sized plasmids seemed to be undergoing faster genetic drift. These plasmids also contained more mobile elements than other replicons. Several toxins and resistance genes were identified, many of which were located on the plasmids.

**Conclusions:**

The completion of the genome of *C. botulinum *group III has revealed it to be a genome with dual identity. It belongs to the pathogenic species *C. botulinum*, but as a genotypic species it should also include *C. novyi *and *C. haemolyticum*. The genotypic species share a conserved chromosomal core that can be transformed into various pathogenic variants by modulation of the highly plastic plasmidome.

## Background

Botulism is a paralytic disease caused by some of the most potent toxins known, the botulinum neurotoxins (BoNTs). The toxins are proteins mainly produced by the species *Clostridium botulinum *but some *Clostridium butyricum *and *Clostridium baratii *strains are also capable of producing BoNTs. Due to their extreme potency, the BoNTs are classified as high risk threat agents for bioterrorism [1]. In addition to causing severe intoxications in humans, BoNT-producing bacteria can also produce serious problems in wild and domesticated animals such as birds, cattle, horses, sheep and minks. Outbreaks with high mortal outcome in waterfowl and poultry have become an increasing environmental and economical problem [2].

*C. botulinum *is an anaerobic, spore-forming Gram-positive bacterium commonly found in soil and aquatic environments [3]. The species *C. botulinum *is divided into four physiological groups (I-IV), which produce BoNTs of seven different serotypes (A-G) [4]. The four groups represent distinct phylogenetic clades and are separated by a distance large enough to merit classification as four separate species [5]. Physiological group I (proteolytic) includes *C. botulinum *strains producing toxins of serotype A, B or F and is mainly associated with human cases. Physiological group II (non-proteolytic) consists of strains producing toxins of serotype B, E or F; these strains also cause human botulism. Group III produces toxins of serotype C or D and is associated with avian and nonhuman mammalian botulism. Genetic studies have shown that strains belonging to physiological group III are more closely related to *C. novyi *and *C. haemolyticum *than to *C. botulinum *serotypes from group I and II [6]. Physiological group IV is a rarer and less characterised group that produces toxin of serotype G.

The neurotoxin genes of *C. botulinum *type C and D are carried by bacteriophages, which express unstable lysogeny and are frequently lost during cultivation. The BoNT prophage propagates in the bacterium as a large plasmid and strains cured from the phage can be reconverted to toxigenic strains by either type C or D phage particles [7]. The distinction between types C and D is not absolute because chimerical sequences exist [8]. In general, the neurotoxin genes analysed from avian isolates comprise parts from both BoNT/C and BoNT/D genes and are referred to as type C/D or D/C [9]. The chimeric types are more lethal to avian species than either type C or D [9,10]. *C. botulinum *types C and D also produce a minor amount of a binary toxin, the C2 toxin, which genes are located on a plasmid [11]. The C2 toxin has a translocation domain as well as an ADP-ribosylating domain that targets actin.

Several completed clostridia genomes have been reported in the last ten years, including *C. difficile, C. perfringens, C. novyi-NT *and *C. botulinum *types A, B, E and F [12-16]. The genome sizes of species in the *Clostridium *genus vary between 2.5 and up to at least 6 Mb and their GC contents are often low. Sequences from three large plasmids from *C. botulinum *group III strains have been released: the BoNT prophage from strain C-Stockholm [17]; the C2 toxin plasmid from strains C203U28 [11] and D-1873; and another large plasmid from strain D-1873. Two draft whole genome shotgun (WGS) assemblies from type D strain 1873 and type C strain Eklund are also deposited in the sequence database.

Here we report and analyse the first complete genomic sequence from a *C. botulinum *group III strain. The sequence comes from a recently isolated *C. botulinum *type C/D strain originating in 2008 from an outbreak in a Swedish poultry farm. We also release three draft genomes: one from a wildfowl outbreak in 2007, another from a poultry outbreak in 2007, and finally the C-Stockholm strain isolated from a mink outbreak in 1949 [18]. The results have revealed a genome with a conserved core that is shaped by plasmids, phages and mobile elements.

## Results and Discussion

### General genome features

**Figure 1:**
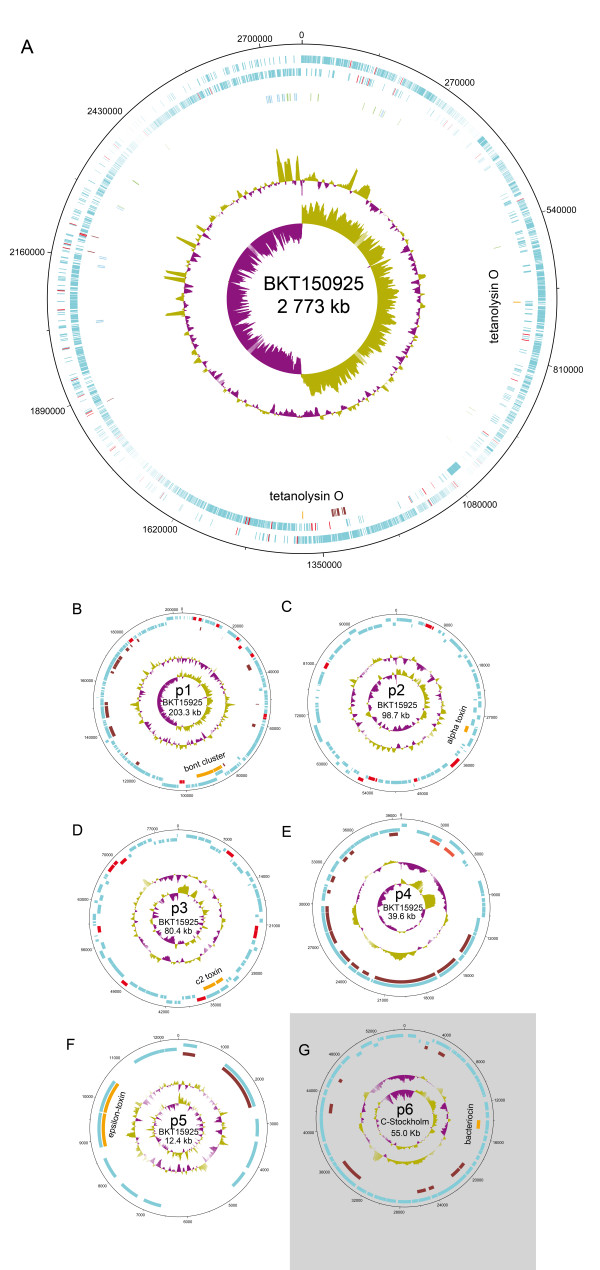
**Circular representation of the replicons characterised from the BKT015925 *C. botulinum *group III genome**. The circles represent (from the outside in): 1, plus strand genes; 2, minus strand genes (mobile elements are marked in red); 3, CDSs coding for phage proteins (brick red), plasmid proteins (light red) and toxins (orange); 4, rRNA genes (blue) and tRNA genes (green). The two inner circles display GC content and GC skew. (A) The chromosome of BKT015925, (B-F) the plasmidome of BKT015925, (G) p6CSt from C-Stockholm.

Features and accession numbers of the replicons from the completed genome of the *C. botulinum *group III strain 08-BKT015925 and approximate data from the draft genomes of strains 07-V891 and C-Stockholm (including the completed plasmid p6CSt) are presented in Table 1. Data from the draft genome of strain 07-BKT028387 (accession number [GenBank:AESB00000000]) are not shown but were very similar to those for BKT015925 with the exception that the BoNT prophage plasmid (p1) was missing due to loss during cultivation. The table also includes features and accession numbers of the genomic sequence of the related *C. novyi-NT *strain (chromosome only) and all available completed *C. botulinum *group III plasmids. In contrast to the previously completed plasmids originating from isolates before 1960, the BKT015925 sequences came from a recent isolate (2008). The *C. botulinum *group III genome was comparably small in relation to other clostridium genomes with a circular chromosome of 2.77 Mbp and a low GC content. Larger deviations of the GC content were only found in association with rRNA operons (Figure 1A). In addition to the chromosome, there were five circular plasmids in the BKT015925 genome, with sizes varying between 12 kb and 203 kb. A sixth plasmid of 55 kb was found in the draft genomes of V891 and C-Stockholm. The plasmid sizes were also verified by pulsed-field gel electrophoresis (PFGE) of S1 nuclease-treated genomic DNA (Additional file 1, Figure S1). The GC contents of all plasmids were similar to that of the chromosome and varied between 26 and 28%. A total of 3026 coding sequences (CDS) were predicted in BKT015925, of which 2546 were located on the chromosome and 480 on the plasmids. In total, 78% of the plasmid content and 86% of the chromosome consisted of CDS. The genes were strongly clustered on the leading strand of the chromosome that was enriched in Gs as seen by the GC skew plot (Figure 1A). The largest plasmid, p1, had a GC skew and a strand-biased distribution of genes similar to the chromosome, indicating two replichores of opposite polarity (Figure 1B). In the second- and third-largest plasmids, p2BKT015925 (p2) and p3BKT015925 (p3), there was less difference in base composition between the strands and no strand-biased gene distribution was observed (Figure 1C-D). The two smallest plasmids/prophages, p4BKT015925 (p4) and p5BKT015925 (p5), and the plasmid found in C-Stockholm, p6CSt (p6), had most of their genes clustered on the same strand (Figure 1E-G). Putative functions were assigned to 2012 (66%) of the chromosomal CDS and to 183 (38%) of the CDS in the total plasmidome. There is often a high number of rRNA operons in clostridium species and the *Clostridium botulinum *group III genome was no exception. We identified 10 rRNA operons and 85 tRNA genes. A noticeably large number of insertion sequence (IS) elements were distributed throughout the genome on both replichores (Figure 1).

**Table 1:** General genomic features of the strains analysed in this study, the *C. novyi-NT *genome and the so far released genomic sequences of *C. botulinum *group III plasmids.

Species and type (strain)	Origin (year of isolation)	GenBank accession number	Replicon	Size (bp)	%GC	CDS (%)
*C. botulinum *C/D	Poultry, Sweden	CP002410	chromosome	2 773 191	28	2546 (86)
(08-BKT015925)	(2008)	CP002411	p1BKT015925^c^	203 287	27	221 (80)
		CP002412	p2BKT015925	98 732	26	103 (75)
		CP002413	p3BKT015925^d^	80 365	27	90 (78)
		CP002414	p4BKT015925	39 648	28	53 (82)
		CP002415	p5BKT015925	12 403	26	13 (61)
			total plasmids	434 435		
			total	3 207 676		
						
*C. botulinum *C/D	Gull, Sweden	AESC00000000	draft genome	~3.1 Mb	28	
(07-V891)	(2007)					
						
						
*C. botulinum *C	Mink, Sweden	AESA00000000	draft genome	~2.7 Mb	27	
(C-Stockholm)	(1949)	AESA00000000	p6CSt	55 058	27	87 (83)
						
						
*C. novyi *A	Gas gangrene	CP00382	chromosome	2 547 720	28	2315 (86)
*(C. novyi-NT)*^b^	(1920)					
						
						
*C. botulinum D*	Pork, Chad	CP001659	pCLG1^d^	107 690	26	124 (78)
(1873)	(1958)	CP001660	pCLG2	54 152	25	47 (25)
						
*C. botulinum C*	unknown	AP010934	pC2C203U28^d^	106 981	26	122 (79)
(C-203U28)^a^						
						
*C. botulinum *C	Mink, Sweden	AP008983	Phage c-Stockholm^c^	185 683	26	198 (83)
(C-Stockholm)	(1949)					

^a ^originally C-203

^b ^originally ATCC 19402

^c^containing the BoNT gene cluster

^d^containing the genes for the C2 toxin

### Phylogenomic comparison

**Figure 2:**
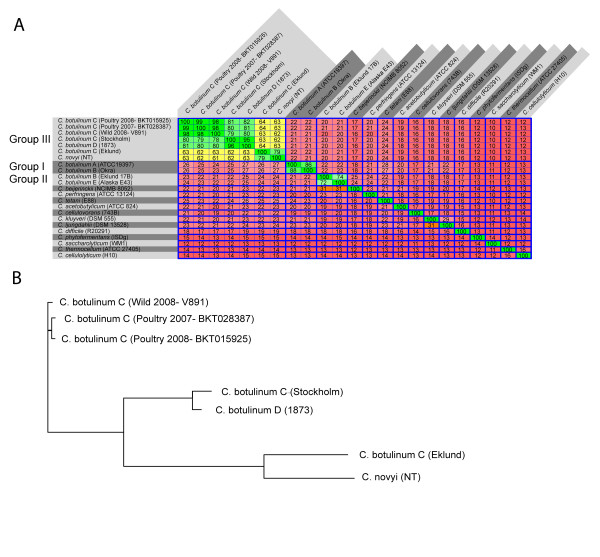
**Phylogenomic analysis of strains of *C. botulinum *group III and in relation to other *Clostridia***. (A) Heat plot (based on the ASC method) showing the results of pairwise average nucleotide distance calculations between the available completed and draft genomes from *C. botulinum *group III and representative genomes from other *C. botulinum *groups and *Clostridium *species.(B) Phylogenetic tree derived from the similarity matrix shown in (A) using the neighbor-joining method.

Results from several methods show that the physiological groups of *C. botulinum *are so distant from each other that they could be considered to be different species [4,19,20]. rRNA comparisons have also shown that *C. novyi *and *C. haemolyticum *are closely related to *C. botulinum *group III [6]. We used the Average Similarity of the Conserved core (ASC) method [20] to compare the pairwise average nucleotide distance between the available completed and draft genomes from *C. botulinum *group III and representative genomes from other *C. botulinum *groups and *Clostridium *species (Figure 2A). The distinct phylogenetic separation of the *C. botulinum *group I-III was obvious. Our results also unequivocally showed that the *C. novyi-NT *genome belongs to the same lineage as the *C. botulinum *C-Eklund genome within *C. botulinum *group III. This provides further evidence that the distinction between *C. botulinum *group III and *C. novyi *does not have a phylogenomic basis. Rather, the distinction has arisen from phenotypic traits linked to clinical symptoms. The *C. botulinum *group III genome sequence contributes further evidence that the current bacterial species classification, at least for some lineages, is inadequate. On one hand, it would be impractical for clinical reasons to change the species name of the physiological groups, where all constituents are causing botulism. Furthermore, it would be confusing to include strains that can cause completely different diseases within the group III species. On the other hand, the current taxonomy conflicts with a phylogenetic concept of the species. We would therefore like to classify *C. botulinum *group III as a dual species. The pathotypic species (pathospecies) *C. botulinum *would include all botulism-causing strains, while the genotypic species (genospecies) *C. novyi sensu lato *would include *C. botulinum *group III, *C. novyi *and *C. haemolyticum*.

Upon comparing the available genomes of group III strains, we found three different clades (Figure 2B). Our recently isolated strains formed one lineage, the D-1873 and the C-Stockholm strains formed a second one and the C -Eklund (type C/D ) together with the *C. novyi*-*NT *strain formed a third lineage. The average genomic variation within group III (*C. novyi sensu lato*) was large, but not exceptionally so.

### Features of the chromosome

The isolates from 2007 and 2008 shared a 98-99% sequence similarity with each other on the nucleotide level in their core genomes, which account for 97-99% of the chromosome. Overall, the BKT015925 chromosome also showed a high resemblance to the *C. novyi *chromosome. The non-conserved regions between BKT015925 and *C. novyi *were scattered throughout the sequence. The BKT015925 chromosome was approximately 9% larger than that of *C. novyi *and the extra genetic material was relatively evenly distributed. Using a BLASTP cut-off at e < 10e-9, 313 proteins were specific for *C. novyi*-*NT *and 511 were specific for BKT015925 (Additional file 2, Table S1). Hypothetical proteins and mobile element-related proteins constituted 58% and 8% of the BKT015925-specific proteins respectively. Among the remaining proteins were components of a phosphotransferase system (PTS) and several large repeat-domain proteins. Most toxin genes were located on the plasmids but there were two tetanolycin O genes found in the chromosome. These tetanolycin genes were also conserved in the *C. novyi-NT *genome. Putative virulence-related genes in the chromosome and plasmidome are summarised in Table 2.

**Table 2:** The distribution of putative genes coding for virulence proteins, antibiotic resistance-conferring proteins, and CRISPR-proteins found in the *C. botulinum *group III completed replicons of the strains analysed in this study.

Feature	**chromosome**^**a**^	**p1**^**a**^	**p2**^**a**^	**p3**^**a**^	**p4**^**a**^	**p5**^**a**^	**p6**^**b**^
**Putative virulence protein**							
Chemotaxis related protein	23		1				
Flagella associated protein	34						
Lipoprotein	15			2			2
Phospholipase	3		1	1	1		
Haemolysin	3		1				
Lipase	1			1			
Peptidase	15						
Protease	9		1		1		
Phage protein	17	23			20	2	10
Toxin	tetanolysin O	bont	alpha-toxin	c2-toxin		epsilon-	bacteriocin
		C/D	(clostripain)			toxin	
							
**Putative antibiotic resistance**							
associated protein							
Drug-export protein	14			1			
Metallo-beta-lactamase	7		2				
Multidrug resistance protein	1						
Microcin self-immunity protein	1						
Tellurium-resistance protein terD	1						
Toxic-anion resistance protein	1						
Penicillin amidase			1				2
							
**CRISPR-associated protein**							
Cas3		1	1				
Cas5		1	1				
Cas6			1				

^a ^replicon from strain BKT015925

^b ^replicon from strain C-Stockholm

**Figure 3:**
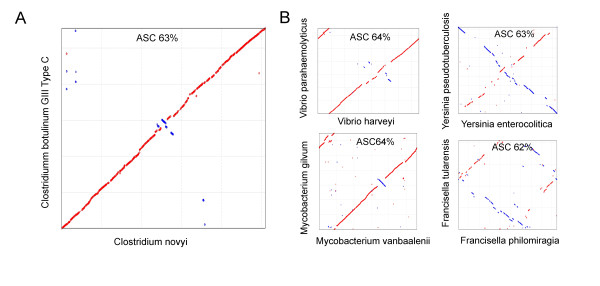
**Genetic synteny and replication-directed translocations visualized by MUMmer alignments**. (A) A comparison of the chromosomes of *C. botulinum *group III (BKT015925) and *C. novyi-NT*. The conserved gene synteny and the low replication-directed translocation activity between the chromosomes are obvious. (B) Four selected pairs of genomes with the same genetic distance to each other as BKT015925 and *C. novyi-NT *show how much the amount of symmetrical inversions can vary.

Bacterial genomes frequently undergo replication-directed translocations. When aligning closely related species these are seen by symmetric inversions around the replication origin [21,22]. There was a high conservation of gene synteny between the BKT015925 and the *C. novyi *chromosomes and only a few symmetric inversions were visible (Figure 3A). The degree of symmetrical inversions between two arbitrary genomes with this particular genetic distance varies tremendously. A few examples are shown in Figure 3B. In conclusion, *C. novyi sensu lato *genomes show a comparably low replication-directed translocation activity.

### Features of the Plasmidome

The five plasmids together comprised 13.5% of the total genomic content of strain BKT015925. To date, this is the largest number of plasmids in any completed clostridia genome. In fact, in all the currently completed microbial genomes, less than 5% of them contain five or more plasmids. The copy number, as determined by coverage, was close to one for the BKT015925 plasmids except for the smallest one, where it was approximately two. By mapping the draft genome reads onto the sequences of plasmids from BKT015925, we concluded that p1-p4 also were present and highly similar (99%, 98%, 97% and 99% nucleotide similarity respectively) in V891. However, the p5 plasmid was absent. By PFGE and analysis of *de novo *contigs unique to V891 and C-Stockholm we could also detect the presence of a sixth plasmid of about 55 kb. This plasmid was completed using the C-Stockholm genome sequence and was therefore called p6CSt. We believe that the plasmids we present here are part of a pan-plasmidome existing in *C. botulinum *group III. Conjugative transfer of BoNT encoding plasmids has previously been reported between strains of *C. botulinum*, and it is likely this mechanism that is responsible for exchange of plasmids between strains [23].

Many of the plasmids contained phage genes and several toxin genes were present in the plasmidome (Table 2). Two of the plasmids also contained clustered, regularly interspaced, short palindromic repeat (CRISPR) regions and genes encoding CRISPR-associated (Cas) proteins, providing acquired immunity against foreign DNA [24]. Typically, CRISPR regions are located on the chromosome and the *C. novyi-NT *genome is no exception with four chromosomal CRISPR regions. Interestingly, CRISPR loci and cas genes were found exclusively on the plasmidome in BKT015925. The number and identity of the spacer sequences on p2 were identical to matches found in the other poultry strain, BKT028387, confirming that the two poultry strains are very closely related.

#### p1BKT015925

**Figure 4:**
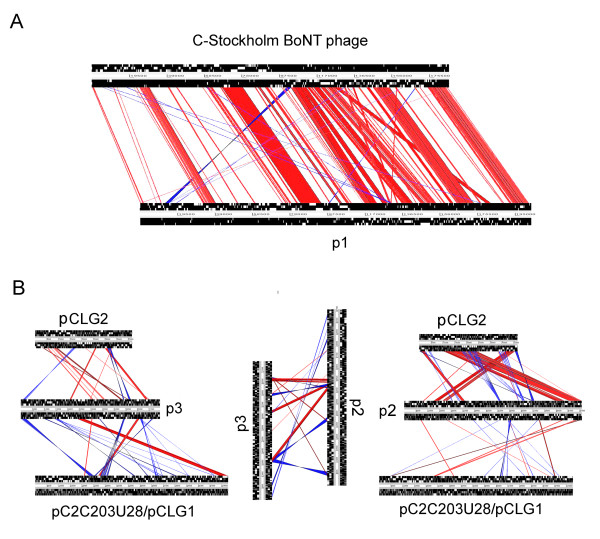
**Comparative analysis of the plasmidome**. Comparisons of the three largest plasmids in BKT015925 with previously completed plasmids from *C. botulinum *group III, performed with Artemis Comparison Tool (ACT). (A) The BoNT-carrying prophage (p1BKT015925) compared to the equivalent, previously released C-Stockholm phage (B) The 99 kb and 80 kb plasmids (p2BKT015925 and p3BKT015925) compared to each other and to plasmids pC2C203U28 (very similar to pCLG1) and pCLG2 from strain C203U28 and D-1873, respectively.

The largest extrachromosomal element, the BoNT-carrying prophage, was found to be a 203 kb circular plasmid in the BKT015925 and V891 genomes. Compared to the BoNT phage from the C-Stockholm stain [17], which has a size of 186 kb, the BKT015925 phage contained 9% more genetic material. A comparison between p1 and phage C-Stockholm is shown in Figure 4A. The synteny of the conserved genetic material was well preserved. For this pair of plasmids, the average nucleotide similarity of the conserved core was in the same range as between the corresponding chromosomes (72% vs. 80%). This indicates that the BoNT prophage and the chromosome are evolving under the influence of similar mechanisms and that they probably have co-existed for a period of time. If genetically diverged strains co-existed in the same habitat, loss of the BoNT prophage in combination with re-infection by one from another strain, could phylogenetically unlink the phage from the chromosome. Our data does not support such scenario but more isolates need to be analysed before this hypothesis can be ruled out. The non-conserved regions, compared to the phage from C-Stockholm, were evenly distributed throughout the replicon and consisted of 110 genes, of which 70% coded for hypothetical proteins, 6% for phage proteins and 8% for mobile elements. Among the remaining proteins specific for p1 were proteins involved in prophage maintenance (death-on curing protein) and defence systems (Cas and abi-alpha proteins). Three CRISPR regions, along with two cas genes, were found in p1, containing 20, 8 and 12 spacers. Only two small CRISPR regions were present in phage C-Stockholm, with 5 and 2 spacers. The majority of the 93 proteins specific for the C-Stockholm phage were also hypothetical proteins (76%), mobile elements (10%) and phage proteins (3%). Phage C-Stockholm and p1 specific proteins are listed in Additional file 3, Table S2.

The progenitor neurotoxin cluster in Group III strains consists of a regulatory protein (BotR), hemagglutinin (HA) proteins and a non-toxic non-hemagglutinin (NTNH) protein. These proteins are suggested to be involved in protection and transportation of the neurotoxin [25]. The complete gene cluster was found in p1, together with the mosaic neurotoxin gene coding for the C-type light (L)-chain and the C/D chimerical heavy (H)-chain. The L-chain (residues 1-444) showed a 98% sequence identity with the corresponding residues in C-Stockholm, but only 48% identity for the same region in type D-1873. By contrast, the C-terminal part of the H-chain (residues 863-1280) shared 42% sequence identity with phage C-Stockholm, but 95% to D-1873. The N terminal part of the H-chain gene showed high similarity to both C and D types and was probably the place where the recombination event once occurred.

#### p2BKT015925

The second-largest plasmid was nearly 100 kb. An interesting feature found in it was an alpha-toxin with conserved domains of the aerolysin superfamily. It showed highest similarity to the alpha-toxin of *C. septicum*, which has both necrotic and haemolytic properties [26]. Another interesting feature was a homologue to the gene coding for clostripain, which was found downstream of the alpha-toxin. This type of cysteine endopeptidase has been found in many clostridia, for example in *C. novyi *and *C. botulinum *group I genomes, but in those cases it was located on the chromosome. The α-clostripain from *C. perfringens *has been suggested to be involved in pathogenesis [27]. Two genes coding for metallo-beta lactamases and one for penicillin amidase were also found in this plasmid; these may contribute to resistance to beta-lactam antibiotics. Three CRISPR regions were found, consisting of 28, 5 and 7 spacers. We also identified three Cas proteins (Cas3, 5 and 6) in proximity to the CRISPR loci.

We compared the p2 plasmid to other group III plasmids (Figure 4B). Some conserved regions were present, especially in the pCLG2 plasmids from *C. botulinum *type D strain 1873. Interestingly, two large, conserved blocks contained all putative virulence factors (except for one protease), two of the Cas genes, and the penicillin amidase gene. Small, scattered, and conserved regions were also shared with pC2C203U28 (and pCLG1) and p3, indicating that genetic crossover events have occurred. This is in contrast to the chromosome and BoNT phage where rearrangements have been few.

#### p3BKT015925

Among the more interesting features of this 80 kb plasmid were two genes coding for putative lantibiotic ABC transporters, and the two genes coding for C2 toxin. Interestingly, the two C2 toxin genes were also found on plasmid pC2C203U28 (and pCLG1), which otherwise shared little resemblance with the p3 plasmid (Figure 4B). The amino-acid sequence of the C2I component shared 96% sequence identity with that of plasmids C-203U28 and pCLG1 and 100% with the C2I sequence of *C. botulinum *strain (C) 2300 [28]. In contrast, the C2II component was more diverged compared to strains 2300 and C-203U28 (88% identity to both). The C-terminal extension found in strain 2300 was also missing. Consistent with previous observations [28], the divergence in the C-terminus of the C2II sequence contributed to most of the differences in sequence identity.

#### p4BKT015925

The majority of the coding sequences in this 40 kb plasmid were phage related, suggesting that this is a prophage. However, it also contained genes coding for plasmid segregation and replication. Interestingly, the GC content and GC skew were different for the prophage and the plasmid-replication part. This could indicate that the prophage may have hijacked a plasmid-replication system.

#### p5BKT015925

The smallest of the plasmids (12 kb), found only in the poultry isolates, contained two putative Epsilon type B toxins. The sequence identity between the two toxin genes was quite low (~40%). A putative homologue was also found in the pCLG1 plasmid but the conservation was low there as well. Two CDS were phage related and all genes but one were located on the same strand. Interestingly, this was a repressor-coding gene that might regulate the transcription of the opposite, gene-dense, strand.

#### p6CSt

Among the CDS identified in this 55 kb plasmid found in C-Stockholm (and V891), eleven were phage related, suggesting that it is a prophage as well. Other genes of interest were two for penicillin amidase and one for a bacteriocin. The latter possibly inhibits growth of competing bacterial strains. All CDS, except for four, were located on the same strand.

### Transposon activity

**Figure 5:**
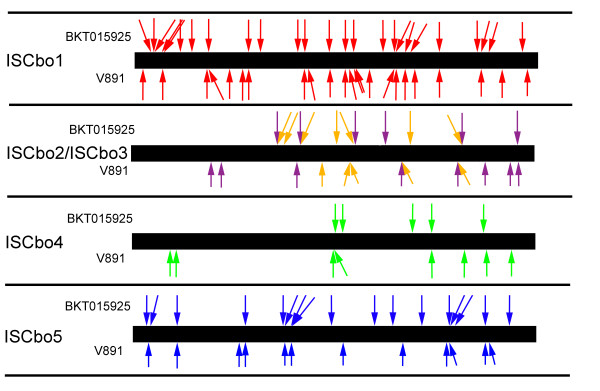
**The transposon activity of high-copy mobile elements**. The mobile elements from the chromosome of BKT015925 and V891 were mapped on a transposon-free consensus chromosome generated *in silico*. The positions of the elements are displayed with arrows. The high-copy elements displayed are ISCbo1 (red), ISCbo5 (blue), ISCbo4 (green), ISCbo2 (brown), and a tandem reverse-transcriptase; ISCbo3 (purple). ISCbo2 has in several cases been inserted into the ISCbo3 element.

It has been found that mobile element abundance in clostridia varies between species and between strains within the same species [13,29,30]. In the *C. botulinum *group III genome BKT015925, many mobile elements were found and some types were present in a high-copy number indicating that the genome is influenced by high transposon activity. To also compare the transposon distribution in the draft genomes (V891 and BKT028387), we mapped reads spanning the insertion points of the elements onto an *in silico *created transposon-free chromosome. The two poultry isolates BKT028387 and BKT015925 had an identical transposon insertion pattern (data not shown). By contrast, the wildfowl isolate V891 had a very different distribution of transposons in its genome (Figure 5). It remains unknown if these elements have moved sporadically during a longer time or if there has been a massive burst of relocations. An overview of the mobile elements present in the chromosomes of BKT015925 and V891 compared to *C. novyi*-*NT *are presented in Table 3.

**Table 3:** Mobile elements identified on the chromosome of strains BKT015925, V891 and *C. novyi-NT*.

IS family	IS element	BKT015925	V891	*C. novyi-NT*
IS6	ISCbo1	25	23	0
IS21	ISCbo2	7	5	0
-	ISCbo3	6	8	0
IS256	ISCbo4	5	8	1
IS1182	ISCbo5	17	12	27

Included among the high-copy mobile elements that showed an elevated transposon activity, there were elements related to the IS6, IS21, IS256 and IS1182 families. There was also a trans-located cassette of two, tandem reverse-transcriptase (RT) genes. In total, the mobile elements accounted for 3.4% of the genetic content of the BKT015925 chromosome and tended to be placed in non-coding regions of both replichores (Figure 1). Except for the IS21-related element, the mobile elements were inserted in intergenic regions, thereby avoiding gene disruptions. IS elements were also found on the three largest plasmids. Two types of elements belonging to the IS200/IS605 family were found exclusively in the plasmidome. In fact, the density of mobile elements was highest on p2 and p3, where 8 and 12% respectively of the total genetic content constituted of such elements. This was interesting because these two plasmids seem to be more genetically dynamic than the chromosome and p1 plasmid. The exchange of plasmids between strains further increases the possibility of horizontal gene transfer facilitated by IS elements.

The IS6 element, ISCbo1, was most related to an IS element, ISCpe7, found in *C. perfringens*. The ISCbo1 elements were found in 25 places (two broken) in the BKT015925 chromosome, of which only eight were on the same place in the V891 draft genome (Figure 5). ISCbo1 elements were also found on p2 and p3. In most cases, the elements were located directly upstream a gene and were surrounded by 8-9-bp non-conserved, direct repeats flanking a 10-bp inverted repeat (Additional file 4, Table S3). Although these elements were not found on the BoNT-carrying prophage in BKT015925, they had previously been identified in a truncated form, between *orfX *and *botR *in the *bont *cluster of *C. botulinum *group I strains. It has been suggested that these elements, together with other closely located transposons, have been the responsible machinery causing horizontal transfer of the *bont *cluster between strains of *C. botulinum *[31].

The IS21-related element, ISCbo2, consisted of two adjacent genes, *integrase *and *istB*. This element was also found in the *C. beijerickii *NCIMB 8052 genome. IstB has an ATP-binding motif, and is thought to be involved in facilitating the contact between target DNA and a transposase, in this case an integrase [32]. The ISCbo2 element was found on six positions on the chromosome of BKT015925, and was also present on the three largest plasmids. It showed a tendency to be incorporated into another mobile element, a tandem reverse-transcriptase (ISCbo3), thereby disrupting its structure. The incorporations were not at the same positions within the RT gene. Small remnants of the IsCbo3 element were found at several places in the genome which could be a consequence of ISCbo2 insertions.

The IS256 family element, ISCbo4, found in the *C. botulinum *group III genome showed high amino-acid sequence similarity (71% identity) to ISCpe3 found in *C. perfringens*. The ISCbo4 element was identified on five locations on the BKT015925 chromosome and on p1-p3. The insertion pattern in V891 was quite different from that in BKT015925 (Figure 5). The element was also found on one location in the *C. novyi-NT *genome.

The IS1182 element ISCbo5 was previously identified (as ISbma2) in numerous copies in the genome of *C. novyi-NT *and *Burkholderia pseudomallei/B. mallei *strains (4 and 48 copies, respectively) [14,33]. Seventeen copies (including one broken) of this element were located on the BKT015925 chromosome, while only twelve copies were found in V891. Six of the copies in V891 were found on the corresponding loci in BKT015925. However, in some cases the insertion sites were slightly altered, which indicates that they have once left and then been reinserted in the same position. There was only one copy within the plasmidome (on p3). All insertion sites except one were composed of inverted repeats forming a hairpin structure. The hairpin structures were exclusively located at the end of genes and most likely act as transcription stop signals. The ISCbo5 element without a surrounding hairpin seems to indicate that it is transcribed together with the upstream gene. It has previously been suggested that such an element is the ancestral one [14]. This specific ISCbo5 element was also broken, indicating it might not be tolerated by the host. The equivalent element (without a surrounding hairpin) in the *C. novyi-NT *genome was located next to a resolvase gene, but in strain BKT015925 it was next to a cysteine desulfurase gene. To test the hypothesis that it is a fused transcript, we amplified the mRNA of the cysteine desulfurase gene by RT-PCR. The gene was transcribed but was not fused with ISCbo5 (data not shown).

Seven different elements belonging to the IS200/IS605 family (ISCbo6-12) were found in p1, and one in p2 (ISCbo13), where five of the IS605 elements were located autonomously and two were found adjacent to IS200 elements. ISCbo7 existed in two copies, both autonomously and adjacent to ISCbo6. Interestingly, this class of elements was only found in the plasmidome. One of the IS200/IS605 elements was highly conserved in the phage C-Stockholm (there called ISCbt4) but it was not located at the same position. By comparing flanking sequences, we located all IS200/IS605 elements to the same positions in the V891 genome, suggesting that these elements are less active than the previously discussed ones.

## Conclusions

The completion of the genome of *C. botulinum *group III has revealed a relatively small chromosome indicating a low tolerance for redundant or excessive genetic material. Acceptance for genes on the lagging strand was also low indicating that high transcription efficiency is crucial for this organism. Although there was a low tendency for rearrangements in the chromosome, size variation between strains indicated that there is an on-going insertion and removal of genetic material. It is probable that the abundant mobile elements are contributing to the genome remodelling process. The *C. botulinum *group III is genetically remote from the types that can cause human botulism, but closely related to *C. novyi*. There are larger differences between certain pairs of *C. botulinum *group III strains than between some group III strains and *C. novyi*. On the basis of these facts, and in analogy with the handling of the *Bacillus cereus *group, we propose a new genotypic name (genospecies), *C. novyi sensu lato*, for *C. botulinum *group III, *C. novyi *and *C. haemolyticum*. However, it is unlikely that the pathotypic name (pathospecies) is going to be changed from *C. botulinum*.

In contrast to the conserved chromosome, we discovered that physiological group III has a remarkably large and variable plasmidome where closely related isolates can have different sets of plasmids. The largest plasmid, the BoNT phage, seemed to obey the same evolutionary rules as the chromosome. In the medium-sized plasmids, and to some extent in the smaller ones, genetic material had been rearranged at a much higher rate and with fewer constrictions. We hypothesise that these plasmids are part of a larger pan-plasmidome and that strains can exchange plasmids.

Mobile elements were abundant also within the plasmidome, where the medium-sized plasmids had the highest transposon density, and they may have been contributing to the mechanism of rearrangements. Interestingly, five out of six toxin types found within the *C. botulinum *group III genome were located in the plasmidome. Our results suggest a model for genome evolution where a conserved core is located in the chromosome while factors affecting pathogenicity are located on a more plastic plasmidome to allow faster environmental adaption without disturbing core functions.

## Methods

### Bacterial strains and preparation of genomic DNA

Three toxigenic *C. botulinum *type C strains (07-V891, 08-BKT015925 and reference strain C-Stockholm) and one non-toxigenic strain (07-BKT028387) were used in this study. Strain V891 was isolated by the authors from a wildfowl herring gull that had died from botulism in Sweden in 2007. Strains BKT015925 and BKT028387 were isolated by the authors from poultry broilers that had died during two separate botulism outbreaks in Sweden in 2008 [2]. Bacterial strains were cultured overnight in anaerobic jars (Merck, Darmstadt, Germany) at 37°C in 9 ml pre-reduced TPGY broth. Cells were harvested by centrifugation at 3000 *g *for 15 min before DNA extraction. Genomic DNA was purified using the DNeasy blood-and-tissue kit (Qiagen, Hilden, Germany). DNA concentration was measured with a NanoDrop 2000 (NanoDrop Technologies, Wilmington, USA) and the DNA was analysed by gel electrophoresis.

### Genome sequencing and assembly

Sequencing of the genomic DNA was done by the whole-genome-shotgun sequencing approach on the Roche Genome Sequencer Titanium system (Roche Applied Science, Mannheim, Germany). The C-Stockholm and BKT028387 strains were sequenced with the FLX chemistry. Approximately 5 μg genomic DNA was subjected to standard 454 shotgun sequencing. The BKT015925 strain was sequenced on a full picotiterplate resulting in 853 000 sequences with an average length of 288 nucleotides. The coverage was approximately 80×. The draft genomes (V891, BKT028387 and C-Stockholm strain) were produced on half a picotiterplate and yielded 385 000, 260 000, and 104 000 sequences respectively.

The sequences were assembled *de novo *using the GS assembler (Newbler, Roche Applied Science). Gaps were closed with help of the Consed package [34]. Sanger sequencing of PCR products and local reassembly were used to resolve gaps, misassemblies and unclear regions. In total, 241 Sanger reads were incorporated into the assembly. Reference guided assemblies were produced with GS mapper or Consed-crossmatch. Genome alignments were made, primarily with Mummer [35] and with the Artemis comparison tool (ACT) [36].

### S1-PFGE

Agarose plugs of strains BKT015925, V891, and C-Stockholm were produced [2] and the gel plugs were incubated with 0.1 units of S1 nuclease (Sigma, St.Louis, MO, USA) for 45 min [37]. The digested DNA was analysed on a 1% agarose gel (Agarose NA; GE Healthcare, Little Chalfont, UK) and electrophoresis was performed in 14°C in HEPES buffer (16 mM HEPES-NaOH, 16 mM sodium acetate, 0.8 mM EDTA, pH 7.5). The settings were 4 V/cm for 26 hours at pulse switch time ramped from 0.5-15 s in a CHEF DRII apparatus (BioRad, Hercules, CA, USA). DNA from *Salmonella Branderup *strain H9812 digested with *Xba*I and a lambda PFG marker (New England BioLabs, Ipswich, MA, USA) were used as size markers.

### Reverse transcription-PCR

Strain BKT015925 was cultured as described and RNA was extracted using Trizol according to the manufacturer's instructions (Invitrogen, Carlsbad, CA, USA). RNA was converted into cDNA using random oligonucleotides and the SuperScript III First-Strand Synthesis System (Invitrogen) according to the manufacturer's procedure. PCR was carried out with gene-specific primers using AmpliTaq Gold polymerase (Applied Biosystems, Foster City, CA, USA), with denaturation at 95°C for 30 s, annealing at 50°C for 30 s and extension at 72°C for 1 min.

### Gene annotation

Genes were defined with Glimmer 3 [38]. The annotation process was handled using Artemis software [39]. Circular plots were drawn with DNAplotter [40]. Further comparisons were made to Swissprot and NCBI non-redundant protein database. Well-conserved genes were automatically annotated but less certain annotations were manually assigned. The length, identity, and coverage of the sequences of the subject versus query were inspected. tRNAs were identified with tRNAscan-SE [41] and rRNA by homology comparison to the *C. novyi-NT *genome. CRISPR loci were found and analysed using CRISPRFinder [42].

### Mapping positions of mobile elements in draft genomes

A mobile-element-depleted genome sequence was generated by removing all high-copy mobile elements from the chromosome of BKT015925. This depleted genome sequence was then used as a consensus for all three genomes (BKT015925, BKT028387 and V891). The positions of the same multi-copy mobile elements in the draft genomes were identified by making a set of GS reference-guided assemblies for the elements and mapping the reads spanning the elements borders back to the mobile-element-depleted genome sequence.

### Phylogenomic comparisons

Phylogenomic distance was measured using the Average Similarity of the conserved Core method (ASC) [20]. When the distance was large and the core was poorly defined on the nucleotide level, total average similarity was used. The dendrogram was created by converting the similarity matrix to a distance matrix and calculating a tree using the neighbor-joining method [43] and PHYLIP 3.67 through the Mobyle platform http://mobyle.pasteur.fr/. The phylogenetic tree was plotted using PhyloDraw 0.82 http://www.bioinformatics.org/wiki/PhyloDraw.

### Nucleotide sequence accession numbers

The sequences and annotations of the 08-BKT015925 chromosome and plasmids have been submitted to GenBank [GenBank:CP002410-CP002415]. The draft genome sequences of C-Stockholm, 07-BKT028387 and V891 were also deposited in GenBank [GenBank:AESA00000000, AESB00000000 and AESC00000000].

## Authors' contributions

BS and HS conceived and designed the study. HS performed most of the gap closure. HS and BS annotated the genome. JW and TH analyzed mobile element insertions. HS performed PFGE and RT PCR. HS and BS performed comparative analysis. BS and HS wrote the manuscript. All authors have read and approved the final manuscript.

## Supplementary Material

Additional file 1 **Figure S1. Pulsed-field gel electrophoresis (PFGE) gel of S1 nuclease treated genomic DNA**. It displays the plasmids of strains C-Stockholm (1), V891 (2) and BKT015925 (3). Lambda marker (M) and *Salmonella Branderup *(SB) DNA digested with *Xba*I, were used as size standards. Click here for file

Additional file 2 **Table S1. List of genes unique to the BKT015925 and *C. novyi-NT *chromosomes in a pairwise comparison**. The Blast cut-off was set to 10e-9. Click here for file

Additional file 3 **Table S2. List of genes unique to the p1BKT015925 and C-Stockholm BoNT phage in a pairwise comparison**. The Blast cut-off was set to 10e-9. Click here for file

Additional file 4 **Table S3. Positions, inverted repeats and direct repeats for the high-copy mobile elements present in the BKT015925 chromosome**. Click here for file
