# Supplementary figures and images for: Clostridium botulinum group III: a group with dual identity shaped by plasmids, phages and mobile elements
Source: BMC Genomics. 2011 Apr 12;12:185. doi: 10.1186/1471-2164-12-185 (PMC3098183; doi:10.1186/1471-2164-12-185)

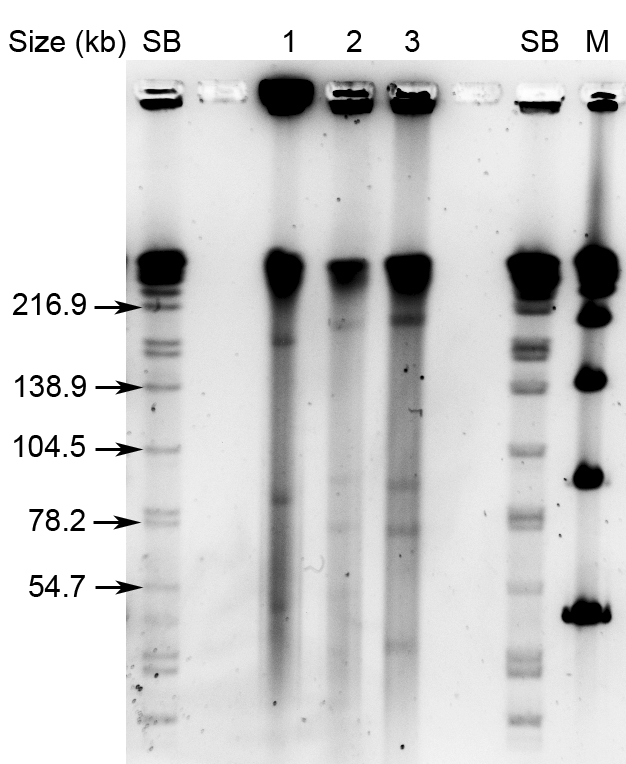

Supplement: Additional file 1 — Figure S1. Pulsed-field gel electrophoresis (PFGE) gel of S1 nuclease treated genomic DNA. It displays the plasmids of strains C-Stockholm (1), V891 (2) and BKT015925 (3). Lambda marker (M) and Salmonella Branderup (SB) DNA digested with XbaI, were used as size standards. [file 1471-2164-12-185-S1.TIFF]
